# Supplementary material for: Assessment of the Phenolic Profiles, Hypoglycemic Activity, and Molecular Mechanism of Different Highland Barley (Hordeum vulgare L.) Varieties
Source: Int J Mol Sci. 2020 Feb 11;21(4):1175. doi: 10.3390/ijms21041175 (PMC7072826; doi:10.3390/ijms21041175)
Supplement: Supplementary file 1 [file ijms-21-01175-s001.pdf]

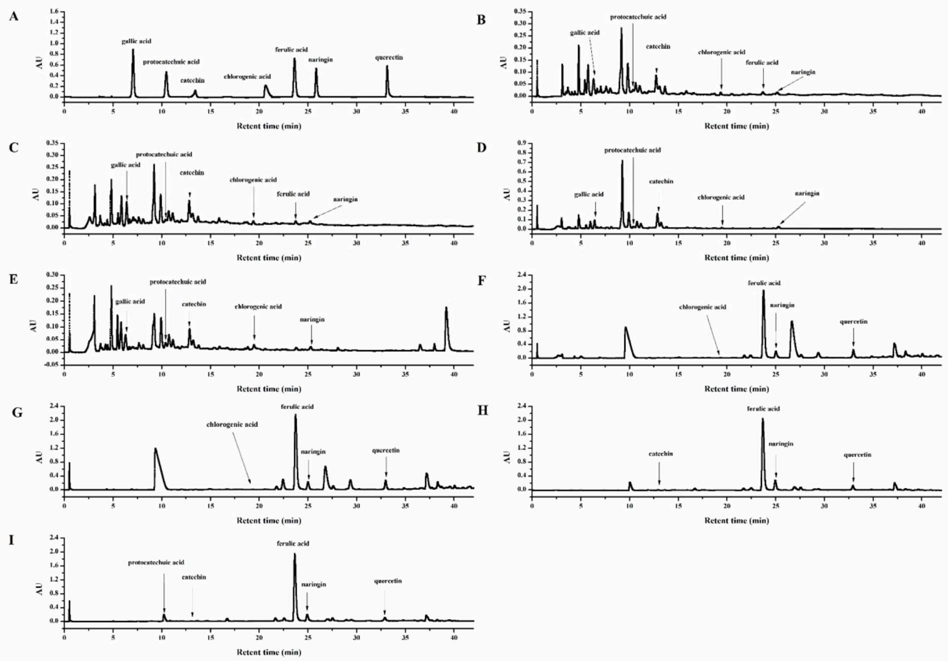

Figure S1. HPLC chromatograms of phenolic profiles of four highland barley varieties: standards of phenolic compounds (**A**), ZQ320 free fraction (**B**), ZQ2000 free fraction (**C**), BQ free fraction (**D**), HQK free fraction (**E**), ZQ320 bound fraction (**F**), ZQ2000 bound fraction (**G**), BQ bound fraction (**H**) and HQK bound fraction (**I**). Flow rate: 0.8 mL/min; Detection is at 280 nm.

Table S1. The limit of detection (LOD) and limit of quantitation (LOQ) of the method, and the spiked recoveries of standards (mean ± SD, n = 3).

Phenolic Compounds	LOD (mg/mL)	LOQ (mg/mL)	Spiked Recovery (%)
Gallic acid	0.013	0.040	102.41 ± 1.37
Protocatechuic acid	0.012	0.036	88.05 ± 2.08
Chlorogenic acid	0.021	0.065	84.85 ± 2.81
Ferulic acid	0.076	0.230	92.09 ± 2.91
Naringin	0.012	0.037	90.94 ± 1.73
Catechin	0.032	0.098	82.86 ± 3.21
Quercetin	0.020	0.061	85.65 ± 2.25

Table S2. Sequences of primer used for RT-qPCR analysis.

Gene Name	Genbank Accession	Primer Sequence (5′-3′)
Homo-IRS-1	NM_005544	GAACTCACTCGGCAGGCACATC
		TGGTGGGTAGGCAGGCATCATC
Homo-PI3K	NM_181523	ACTGAAGCAGATGTTGAACAAC

		CATCGATCATTTCCAAGTCCAC
Homo-Akt	NM_001243028	GCGGAGCAGGGCGTTTCAC<br>AACCACAGGAGGGAGGCAACC
Homo-GSK3*β*	NM_002093	CGTCCGTGATTGGCTCCGTTC<br>CTCGTCCTCCACCTCCTTCCTC
Homo-G6Pase	NM_000151	GTAGGTGGTGGAGGGCAGGAG<br>GGTGTGGGAGCGGGCTAGG
Homo-PEPCK	NM_002591	CATCCCAACTCTCGATTTTGTG<br>TTCCCAGAAGTCCTTTGTGTTC
Homo-GYS2	NM_021957	GTGACAGCAGCCAGCAGTAGC<br>TGAGAGCCCAACACCCACCAG
Homo-GLUT4	NM_001042	CTGAAGGATGAGAAGCGGAAG<br>TCGAAGATGCTGGTCGAATAAT
Homo-*β*-actin	NM_001101	ATCGTCCACCGCAAATG<br>CTGTCACCTTCACCGTTCC
